# Comparison of Anthropometric Indicators That Assess Nutritional Status From Infancy to Old Age and Proposal of Percentiles for a Regional Sample of Chile

**DOI:** 10.3389/fnut.2021.657491

**Published:** 2021-12-24

**Authors:** Rossana Gómez-Campos, Rubén Vidal-Espinoza, Anderson Marques de Moraes, Evandro Lázari, Cynthia Lee Andruske, Luis Castelli Correia de Campos, Luis Urzua-Alul, Wilbert Cossio-Bolaños, Marco A. Cossio-Bolanõs

**Affiliations:** ^1^Departamento de Diversidad e Inclusividad Educativa, Universidad Católica del Maule, Talca, Chile; ^2^Facultad de Educación, Universidad Católica Silva Henriquez, Santiago, Chile; ^3^Faculty of Physical Education, Pontifical Catholic University of Campinas, Campinas, Brazil; ^4^Faculty of Applied Sciences, UNICAMP, Limeira, Brazil; ^5^Centro de Investigación CINEMAROS, Arequipa, Peru; ^6^Departamento de Ciencias de la Educación, Universidad de Bio Bio, Chillán, Chile; ^7^Escuela de Kinesiología, Facultad de Salud, Universidad Santo Tomás, Viña del Mar, Chile; ^8^Escuela de Posgrado, Universidad Privada San Juan Bautista, Lima, Peru; ^9^Departamento de Ciencias de la Actividad Física, Universidad Católica del Maule, Talca, Chile

**Keywords:** nutritional state, percentiles, children, adolescents, adults

## Abstract

**Objectives:** Anthropometric variables are used to evaluate health, dietary status, disease risks, and changes in body composition. The purpose of this study was to compare weight, height, and Body Mass Index (BMI) with American references from the National Center for Health Statistics (NCHS-2012), using BMI and Tri-Ponderal Mass Index (TMI) to propose percentiles for evaluating nutritional status of children, adolescents, and adults, ages 5–80 years old.

**Methods:** A descriptive cross-sectional study was conducted in 15,436 (8,070 males and 7,366 females) children, youths and adults in the Maule region (Chile). The age range ranged from 5.0 to ~80 years of age. Weight and height were assessed. Body mass index BMI and tri-ponderal mass index (TMI) were calculated. The LMS method was used to generate percentiles.

**Results:** The results illustrated that children were heavier and had more BMI during childhood compared to the NCHS references. During adolescence, reference values were greater until approximately ages 70–79. For height, children were relatively similar to those of the NCHS references, but during adolescence, differences became evident. Adolescence until approximately age 80, the population showed lower values for height. Percentiles were calculated using BMI and TMI by age range and sex. Differences occurred between the American NCHS references and the population with regard to the anthropometric variables of weight, height, and in BMI.

**Conclusion:** Discrepancies with the American NCHS reference were verified in the anthropometric variables of weight, height and BMI. Reference percentiles of BMI and TMI were developed for the evaluation of the nutritional status of the regional population of Maule (Chile). Its use is suggested in clinical and epidemiological contexts.

## Introduction

Access to adequate nutrition is a critical component necessary for a human being during all stages of life. The human body needs a proper well-balanced nutritional diet to meet the body's requirements and maintain basic body physiology (1).

Currently, the primary nutritional problems that affect children, adolescents, youth adults, adults, and older adults include malnutrition, underweight, overweight, and obesity. In general, malnutrition (for underweight or overweight) affects future physical development of a human being. Malnutrition reduces the capacity to work, resistance to physical exertion, and the ability to concentrate. Over eating predisposes individuals to chronic diseases, such as diabetes, cardiovascular diseases (2), among others.

Many regions of the world are actually experiencing the double burden of nutritional problems (1). This translates into a public health threat for countries with low and medium incomes (3).

In this sense, epidemiological surveys worldwide need to adopt strategies that help evaluate nutritional conditions using diverse methods. In general, a person's nutritional status can be measured with the popular method known as ABCDE (4). A represents anthropometric measurements, such as height and weight; B, biochemical parameters, such as serum albumin level and hemoglobin count; C, clinical assessment, which includes an assessment of functional, social and mental status, medical history and physical examination; and D, dietary history, such as supplement use and adequacy of diet; and E, evaluation.

In fact, to assess the nutritional status of large populations, one of the most widely used techniques is anthropometry. This technique is considered to be a key component in the evaluation of the nutritional status of children and adults (5). In addition to its long historical tradition, it is an inexpensive and non-invasive method that provides detailed information about the different body structures, especially muscular and fat components (6). Furthermore, it is considered to be a universally applicable method for evaluating body size, composition, and proportion (7).

To understand the nutritional condition and/or goals of an individual population, it is necessary to assess anthropometric variables. For example, when these are applied to children, these help show the general health status, dietary adequacy, and help monitor growth and development over time. However, when applied to adults, anthropometric variables are used to evaluate health, dietary status, disease risks, and analysis of possible changes in body composition during adulthood (8).

In this context, an anthropometric evaluation of the nutritional status of the regional population of Maule in Chile is important, since it merits a formal and consistent means that contributes to risk assessment, monitoring of nutritional changes and possible comparisons with national and international studies, since this information could provide greater opportunities for comparison, not only with other regions of the country, but also with neighboring countries in South America.

Actually, a number of institutions around the world have been interested in proposing curves for evaluating physical growth and nutritional status in children and adolescents (9–11). However, very few studies exist that evaluate the trajectory of the nutritional status of individuals from birth to old age, except for population studies from the National Center for Health Statistics (NCHS) form the United States (8, 12). The NCHS has developed anthropometric references for children and adults, spanning the entire lifecycle.

These references, in general, are used by a number of countries for assessing physical growth trajectories and the nutritional condition of children, adolescents, youth, and adults. Only recently in Chile, percentiles have been developed to evaluate physical growth and body adiposity in children and adolescents (13, 14). However, to date, these references are incomplete because they do not take into account other life stages, such as young adulthood, adulthood, and old age.

As a result, the references, in general, use anthropometric indexes to assess the nutritional status of populations. Therefore, they appear to remain the most commonly used tools for public health and, particularly, in developing countries for evaluating and monitoring physical growth and nutritional status (15).

Developing references based on Body Mass Index (BMI) and the Tri-Ponderal Mass Index (TMI) for a regional population in Chile, spanning all stages of life, could have implications relevant for evaluating nutritional status and for developing public policies. In addition, they could be important for prevention and control for the health status for the population of the Maule Region of Chile.

As a result, for decades, BMI has been recommended and considered as the classic indicator for detecting underweight, overweight, and obesity in diverse populations around the world (16, 17). However, recently, TMI (weight divided by height cubed) has been considered as an indicator of satisfactory body adiposity in relation to BMI (17, 18). Thus, its inclusion in the nutritional state is relevant.

From this perspective, in this study, we hypothesized that it is possible that different anthropometrics exist between the CDC-2012 references and the regional study of the Maule. Therefore, these discrepancies could give rise to the proposed percentiles for assessing the nutritionals state from childhood to old age. Thus, the first initial objectives for this study were to compare weight, height, and BMI with the American references from the NCHS-2012 and to propose percentiles to assess the nutritional statues using BMI and TMI in a regional sample of individuals between the ages of 5–80 years old in the Maule Region of Chile.

## Materials and Methods

### Type of Study and Sample

A descriptive cross-sectional study was carried out with 15,436 (8,070 males and 7,366 females) children, adolescents, and adults from the Maule Region (Chile). The sample selection was non-probabilistic (quotas). This sample is 2.2% of the urban population of the Maule region. In general, according to the latest report of the Chilean Ministry of Social Development (19), 709,418 inhabitants (341,700 men and 367,718 women) ranging from 5 to 80 years old live in the urban area of the Maule region. All subjects recruited volunteered from public schools, universities (public and private). Youth, adults, and older adults volunteered from social programs offered by the Municipality of Talca (Maule Region of Chile). Age ranged from 5.0 to 80 years old. Maule is the seventh region of Chile. Talca is the capital city. The region consists of four provinces: Cauquenes, Curicó, Linares, and Talca. The Maule Region is located 230 km south of Santiago, the capital of Chile. The region is 102 meters above sea level.

According to the United Nations Development Program (20), the Human Development Index (HDI) of Chile highlighted that for 2018, the HDI was 0.847. Life expectancy is 80 years of age, and the average education is ~16.5 years. In the Maule Region, the HDI was 0.872. Table 1 shows some social development indicators as described by the Ministry of Social Development Chile (19). The indicators in Table 1 illustrate the small differences between the Maule Region (Talca) with the capital (Santiago) and the northern regions of Arica and Parinacota and the southern region of Magallanes.

**Table 1:** Social development indicators of the Maule Region compared to Santiago (Chile).

**Indicators**	**Arica and Parinacota (Northern Region)**	**Santiago (Capital)**	**Maule (Central Region)**	**Magallanes (South Region)**	**Chile**
**Education**					
Net rate of pre-school attendance (0–5 years)	54.0%	50.4%	50.7%	56.1%	50.3%
Net rate of elementary school attendance	91.5%	91.1%	92.8%	94.6%	91.5%
Net rate of secondary school attendance	74.3%	83.8%	72.6%	71.0%	73.6%
Net rate of higher education attendance	38.5%	39.1%	33.2%	41.0%	37.4%
Average number of years of education	11.4%	~11.6	~9.8	11.2%	11.0%
**Employment**					
Participation rate in the labor market	59.9%	63.2%	55.7%	60.4%	58.3%
Employment rate	54.9%	58.9%	51.8%	54.0%	54.0%
Unemployment rate	8.3%	6.9%	6.9%	7.5%	7.5%
**Health**					
Health care (State)	76.3%	71.0%	86.2%	69.9%	77.3%
Health care (Private)	10.9%	21.3%	5.9%	15.9%	15.1%
No health care	3.9%	3.3%	3.0%	3.0%	3.1%

All school children, university students, and adults providing informed consent and completing the evaluations of the anthropometric variables were included in the study. Nevertheless, subjects excluded were those of another nationality as well as those not consenting to the anthropometric assessments. This study was carried out according to the Declaration of Helsinki for Human Subjects and the suggestions from the Ethics Committee from the Universidad (UA, 238/2014). Parents and/or teachers signed the informed consent forms for minors under age 18, and those 18 and older (>18 years old) signed the informed consent themselves. The evaluations were conducted by experienced professionals trained in the necessary evaluation procedures (four professionals).

### Procedures

Data, such as age, sex, school, university, and workplace, were recorded in individual files. All anthropometric variables were evaluated in the school, university, work place, and laboratory from the Universidad. One of the researchers coordinated a work team consisting of 4 experienced evaluators to collect data.

The evaluations were carried out during 2015, 2016, 2017, and 2018 from 8:30 a.m. to 13:00 p.m. and 15:00 to 18:00 p.m. Furthermore, the assessments took place from Monday to Friday during the months of April to June and August to November during each year of the study.

The evaluations of the anthropometric variables of weight and height were carried out according to Ross and Marfell-Jones' (21) standardized protocol. During the assessments, subjects wore the least amount of clothing possible (shorts, T-shirt, and barefoot). To assess body weight (kg), an electric scale (Tanita, Glasgow, United Kingdom, Ltd), with a scale of 9 to 150 kg and an accuracy of 100 g. was used. Standing height was measured with a portable stadiometer (Seca Gmbh & Co. KG. Hamburg, Germany), with an accuracy of 0.1 mm. based on the Frankfurt Plane. Body Mass Index (BMI) was calculated using the following formula: BMI = weight (kg)/height^2^ (m), and the TMI = weight (kg)/height^3^ (m).

To ensure quality control of the anthropometric variables, 10% (1,550 subjects) of the sample was evaluated twice. The intra- and inter-evaluator TEM technical measurement error showed values between 1 and 2%.

Using the 50th percentile, the anthropometric variables (weight, height, and BMI) were compared with the curves of the U.S. National Center for Health Statistics (8). They were also compared with the World Health Organization (WHO) BMI medians (11).

### Statistics

The data for the normal distribution of the data was determined using the Kolmogorov-Smirnov test. Differences between the sexes were obtained using the *t* test for independent samples. To fit the data (weight and height), the best model was selected based on the *R*^2^, residual standard error (RMSE) and significance. Different regression analysis models were used, with the eighth degree cubic polynomial model being the most appropriate for weight and height. The calculations and graphs of the curves were obtained by means of the computer program implemented in the R. The proposed percentiles were created to evaluate BMI and PI: p3, p5, p10, p15, p25, p50, p75, p85, p90, p95, and p97. The LMS method was also used to create the percentiles (L: Box-Cox coefficient; M: median; S: coefficient of variation) (22). Graphs were created with *Chart Maker* version 2.3 software (23). Differences between the percentiles were performed using the fraction: 100 log (reference percentile/calculated percentile). For all cases <0.05 was adopted. Calculations were performed with Excel and SPSS 18.0.

## Results

The anthropometric variables and body indices from age 5.0 to >80 years are shown in Table 2. For weight and height, there were no significant differences between the age ranges 5.0–7.9 years and 11.0–13.9 years. However, in males, from the age range of 14.0–16.9 to age 80 years, they were heavier and taller compared to females. There were no significant differences in both sexes for BMI in the age ranges from 5.0–7.9 to 50.0–59.9 years. However, there were differences from the age range 60.0–69.9 to 80 years, where females showed a higher BMI than males. For the TMI, no differences were observed in the age ranges 5.0–7.9, 8.0–10.9 and 40.0–49.9 years. However, for the rest of the age ranges, women presented higher values than males.

**Table 2:** Anthropometric characteristics of the sample studied.

**Age range**	***n***	**Weight (kg)**		**Height (cm)**		**BMI (kg/m** ^**2**^ **)**		**TMI (kg/m** ^**3**^ **)**	
		**X**	**SD**	**X**	**SD**	**X**	**SD**	**X**	**SD**
**Males**									
5–7.9 years	634	26.37	6.33	121.03	8.33	17.84	2.87	14.78	2.29
8–10.9 years	1,137	36.98	9.64	136.11	8.36	19.73	3.63	14.49	2.49
11–13.9 years	1,340	50.04	12.00	153.51	9.74	21.07	3.85	13.76^*^	2.55
14–16.9 years	1,445	66.02^*^	12.94	169.14^*^	7.67	23.02	4.02	13.64^*^	2.53
17–19.9 years	1,641	71.75^*^	12.13	172.03^*^	6.57	24.24	3.91	14.12^*^	2.45
20–29.9 years	1,058	77.20^*^	12.88	173.07^*^	6.67	25.72	3.71	14.88^*^	2.20
30–39.9 years	111	81.38^*^	12.25	175.34^*^	9.45	26.39	2.77	15.09^*^	1.74
40–49.9 years	64	85.69^*^	18.09	169.95^*^	4.79	29.58	5.51	17.40	3.13
50–59.9 years	117	81.36^*^	13.22	169.49^*^	6.87	28.30	4.12	16.73^*^	2.56
60–69.9 years	272	78.45^*^	13.72	167.44^*^	7.02	27.98^*^	4.74	16.76^*^	3.05
70–79.9 years	203	76.50^*^	10.86	165.61^*^	6.85	27.92^*^	3.90	16.91^*^	2.66
>80 years	48	70.78^*^	8.29	163.34^*^	6.59	26.54^*^	2.93	16.29^*^	2.09
Total	8,070	60.09^*^	20.71	159.13^*^	18.50	22.92^*^	4.74	14.43^*^	2.62
**Females**									
5–7.9 years	659	25.59	5.47	120.33	7.18	17.56	2.69	14.62	2.22
8–10.9 years	789	36.64	9.49	136.40	9.00	19.47	3.51	14.29	2.44
11–13.9 years	1,082	51.76	11.92	153.40	7.60	21.87	4.24	14.27	2.78
14–16.9 years	918	60.19	11.16	159.01	6.20	23.79	4.16	14.99	2.74
17–19.9 years	1,105	61.69	11.56	159.91	5.92	24.09	4.08	15.08	2.60
20–29.9 years	949	64.10	11.32	161.23	5.91	24.64	4.02	15.31	2.60
30–39.9 years	178	71.59	14.48	160.16	7.09	27.91	5.25	17.49	3.54
40–49.9 years	190	69.40	13.17	157.50	6.55	27.98	5.04	17.81	3.37
50–59.9 years	292	71.07	12.28	156.66	5.91	29.00	5.04	18.56	3.47
60–69.9 years	701	69.81	11.70	155.20	7.14	29.04	4.84	18.79	3.46
70–79.9 years	433	67.82	11.69	152.95	6.59	28.98	4.49	18.99	3.12
>80 years	70	64.23	12.75	152.50	7.00	27.62	5.13	18.16	3.58
Total	7,366	56.41	17.62	151.84	13.95	23.90	5.45	15.73	3.29

*X, average; SD, standard deviation; BMI, Body Mass Index; TMI, tri-ponderal mass index*.

* *Significant difference in relation to females*.

**Figure 1:**
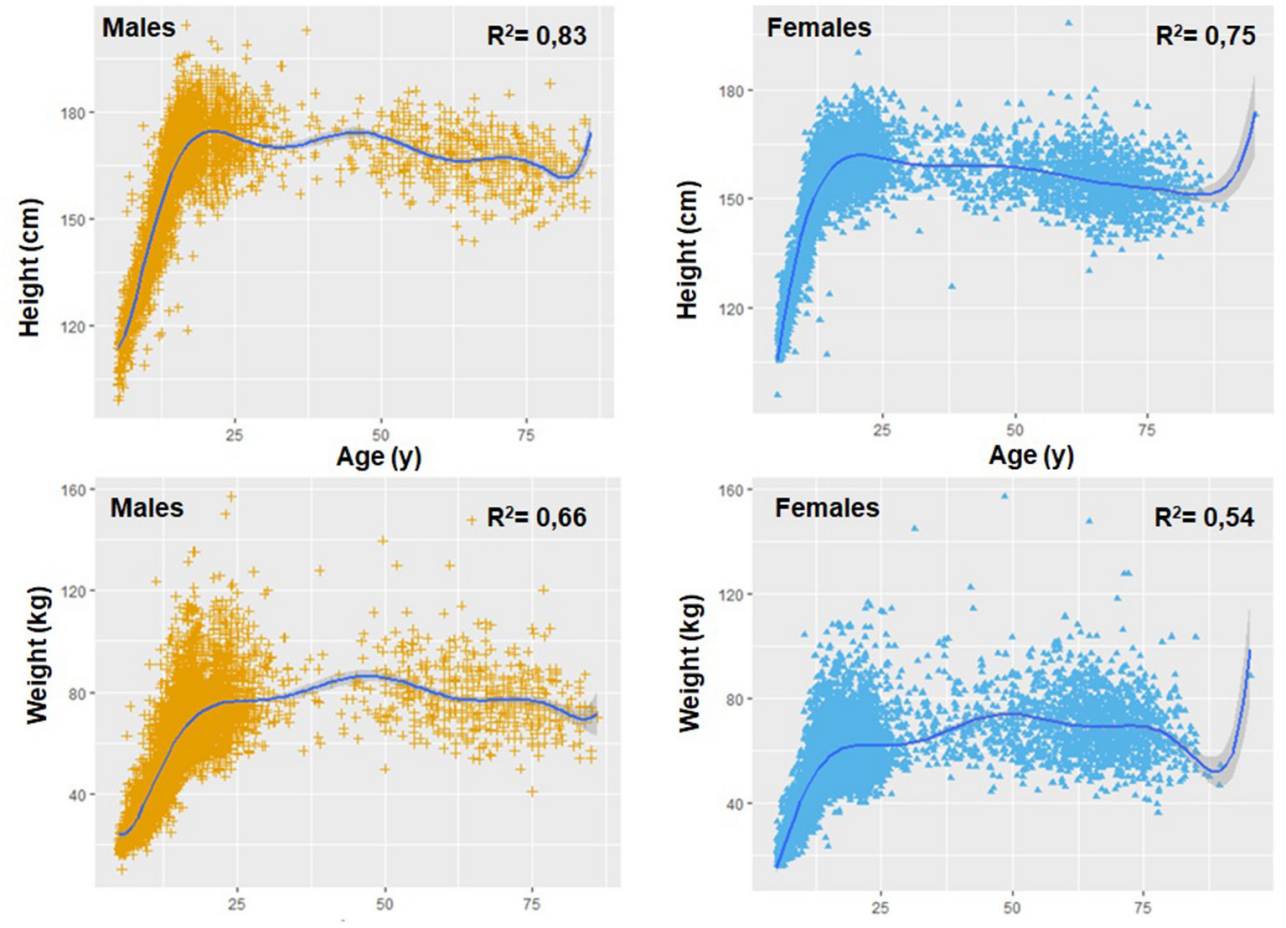
Polynomial relationship of age with weight and height in both sexes.

The fit of the data for the weight and height curve showed a polynomial regression of degree 8. This polynomial regression model indicated a significant relationship between chronological age with weight and height in both sexes (see Figure 1). The fit of the data for the height curve was *R*^2^ = 0.84 for males and *R*^2^ = 0.75 for females, while for the weights, these values were lower (*R*^2^ = 0.66 for males and *R*^2^ = 0.54 for females). In general, the adopted model (grade 8) reflected a good fit, with RMSE values ranging from 6.9 to 6.3 for height and from 11.5 to 10.7 for body weight.

**Figure 2:**
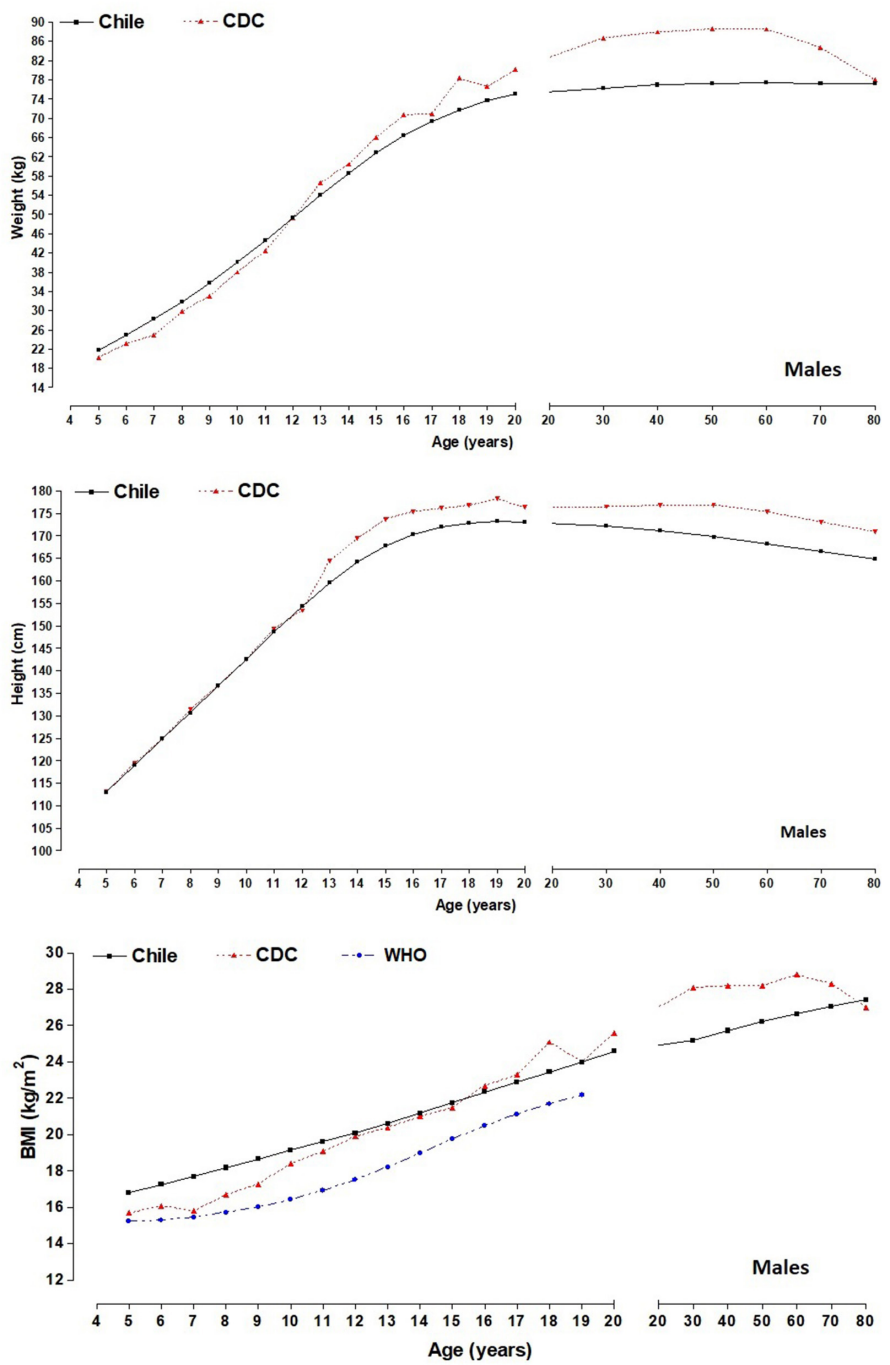
Comparison of differences (50th percentile) of anthropometric variables (weight and height) between the Maule regional sample and the CDC-2012; and differences in BMI between the study sample, the CDC-2012 and the WHO reference for males.

**Figure 3:**
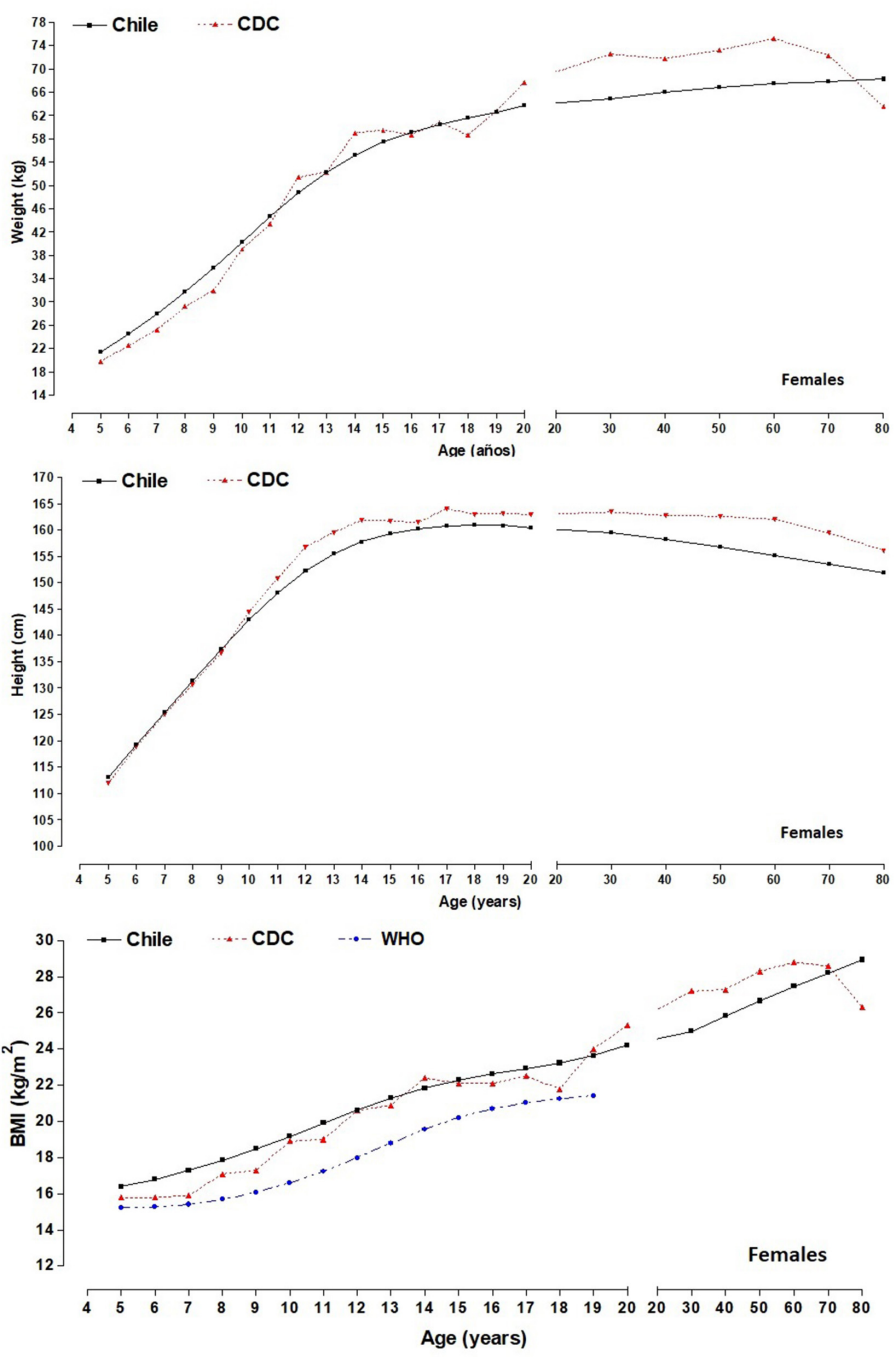
Comparison of differences (50th percentile) of anthropometric variables (weight and height) between the Maule regional sample and the CDC-2012; and differences in BMI between the study sample, the CDC-2012 and the WHO reference for females.

Figures 2, 3 illustrate the comparisons of the anthropometric variables of weight, height, and BMI of the regional population of the Maule with the American references of the CDC-2012 (50th percentile). With regard to body weight, from age 5.0 to 11.9 years old, children from the Maule Region showed greater weight than those for the CDC-2012 references (for males, from ~0.09 to ~5.40 kg and for females, from ~1.28 to ~5.13 kg). Then, from 12.0 to 80 years old, the CDC-2012 references presented higher values in both sexes (for males, from ~0.45 to ~5.93 kg and for females, from ~0.05 to ~4.80 kg), except for females at age 80 where they showed values higher of ~3.10 kg.

For height, the growth patterns for both sexes were similar: for males until age 12.9 and for females until 11.9 years old. Then, the CDC-2012 reference values were higher than their Maule counterparts up to age 80. The differences for males were from ~0.82 to ~1.80 cm, and for females, it was from ~0.32 to ~1.89 cm.

With regard to BMI, the children of both sexes from the Maule showed higher values from age 5.0 to 11.9 years old. Differences for males were greater from ~1.12 to ~4.93 kg/m^2^ while for females, differences began from ~0.68 to ~3.66 kg/m^2^. Then, from 12.0 years old to age 17.9, the values were relatively similar, ranging for males, from ~0.41 to ~0.45 kg/m^2^ and for females, from ~0.01 to ~0.97 kg/m^2^. Commencing at age 18.0 until 70–79.9 years old, the CDC-2012 values were higher for both sexes. For example, the values for male were higher from ~0.03 to ~4.73 kg/m^2^ and for females, from ~0.61 to ~3.66 kg/m^2^. Finally, at age 80 years old. BMI values for both sexes were greater (in males, ~0.64 kg/m^2^ and in females, ~4.09 kg/m^2)^, vs. the CDC-2012 reference values.

In relation to comparisons of BMI among children and adolescents in Maule with the WHO 50th percentile, we can highlight that the WHO reference values reflect lower medians in both sexes (in males from ~1.5 to 2.7 kg/m^2^ and in females from ~1.1 to 2.6 kg/m^2^) and from 5 to 19 years of age.

Tables 3–6 show the values for BMI and TMI from age 5.0 to 80 years of age. These results are presented in percentiles based on age ranges and sex (p3, p5, p10, p15, p25, p50, p75, p85, p90, p95, and p97). The curve for BMI values for both sexes increased linearly from age 5.0 to age 80. The BMI in both sexes was relatively similar and was slightly different in 50th percentile (~0.10 to ~0.80 kg/m^2^) from ages 5.0–5.9 to 60.0–69.9 years of age. However, in the last two age ranges (70.0–79.9 and >80 years), females showed higher values when compared to males, between ~1.2 to ~1.5 kg/m^2^.

**Table 3:** Percentiles for evaluating BMI from 5 to 80 years old based on age range for males.

**Age**	**n**	**L**	**M**	**S**	**P3**	**P5**	**P10**	**P15**	**P25**	**P50**	**P75**	**P85**	**P90**	**P95**	**P97**
5.0–5.9	149	0.74	16.8	0.17	11.6	12.2	13.2	13.9	14.9	16.8	18.8	19.9	20.7	21.8	22.5
6.0–6.9	206	0.46	17.3	0.17	12.1	12.7	13.7	14.3	15.3	17.3	19.3	20.5	21.3	22.6	23.4
7.0–7.9	279	0.19	17.7	0.17	12.7	13.2	14.1	14.8	15.7	17.7	19.9	21.1	22.0	23.4	24.3
8.0–8.9	377	−0.1	18.2	0.17	13.2	13.7	14.6	15.2	16.2	18.2	20.4	21.8	22.7	24.2	25.2
9.0–9.9	302	−0.3	18.7	0.17	13.7	14.2	15.1	15.7	16.7	18.7	21.0	22.4	23.4	25.1	26.2
10.0–10.9	458	−0.5	19.2	0.17	14.2	14.7	15.6	16.2	17.1	19.2	21.6	23.0	24.1	25.9	27.1
11.0–11.9	524	−0.7	19.6	0.17	14.7	15.2	16.0	16.6	17.6	19.6	22.1	23.6	24.8	26.7	28.0
12.0–12.9	396	−0.8	20.1	0.17	15.2	15.7	16.5	17.1	18.1	20.1	22.6	24.2	25.4	27.4	28.9
13.0–13.9	420	−1	20.6	0.16	15.8	16.2	17.0	17.6	18.6	20.6	23.2	24.8	26.1	28.2	29.7
14.0–14.9	457	−1.1	21.2	0.16	16.3	16.8	17.6	18.2	19.1	21.2	23.8	25.4	26.7	28.9	30.6
15.0–15.9	524	−1.2	21.8	0.16	16.9	17.4	18.2	18.8	19.7	21.8	24.4	26.1	27.4	29.7	31.3
16.0–16.9	464	−1.3	22.4	0.15	17.5	18.0	18.8	19.3	20.3	22.4	25.0	26.7	28.0	30.3	32.0
17.0–17.9	777	−1.3	22.9	0.15	18.0	18.5	19.3	19.9	20.8	22.9	25.5	27.3	28.6	30.9	32.6
18.0–18.9	397	−1.4	23.5	0.15	18.5	19.0	19.8	20.4	21.4	23.5	26.1	27.8	29.1	31.4	33.1
19.0–19.9	467	−1.4	24	0.14	19.1	19.5	20.4	21.0	21.9	24.0	26.6	28.4	29.7	31.9	33.6
20.0–29.9	1,058	−1.3	24.6	0.14	19.6	20.1	20.9	21.5	22.5	24.6	27.2	28.9	30.3	32.5	34.2
30.0–39.9	111	−1.3	25.2	0.14	20.1	20.6	21.5	22.1	23.0	25.2	27.8	29.6	30.9	33.1	34.7
40.0–49.9	64	−1.3	25.7	0.14	20.6	21.1	22.0	22.6	23.6	25.7	28.4	30.1	31.4	33.6	35.2
50.9–59.9	117	−1.3	26.2	0.14	21.0	21.5	22.4	23.0	24.0	26.2	28.9	30.6	31.9	34.1	35.7
60.9–69.9	272	−1.2	26.7	0.13	21.4	21.9	22.8	23.4	24.5	26.6	29.3	31.0	32.3	34.5	36.1
70.9–79.9	203	−1.2	27	0.13	21.7	22.3	23.2	23.8	24.8	27.0	29.7	31.4	32.7	34.8	36.4
>80 years	48	−1.2	27.4	0.13	22.1	22.6	23.5	24.2	25.2	27.4	30.1	31.8	33.1	35.2	36.7

*LMS, least-mean-square algorithm; L, Box-Cox coefficient; M, median; S, coefficient of variation*.

**Table 4:** Percentiles for evaluating BMI from 5 to 80 years old based on age range for females.

**Age**	***n***	**L**	**M**	**S**	**P3**	**P5**	**P10**	**P15**	**P25**	**P50**	**P75**	**P85**	**P90**	**P95**	**P97**
5.0–5.9	137	−0.28	16.40	0.16	12.2	12.7	13.4	13.9	14.7	16.4	18.3	19.5	20.3	21.6	22.5
6.0–6.9	220	−0.35	16.81	0.16	12.6	13.0	13.7	14.3	15.1	16.8	18.8	20.0	20.9	22.3	23.3
7.0–7.9	302	−0.43	17.29	0.17	12.9	13.4	14.1	14.6	15.5	17.3	19.4	20.7	21.6	23.1	24.1
8.0–8.9	267	−0.50	17.85	0.17	13.3	13.8	14.6	15.1	16.0	17.8	20.0	21.4	22.4	24.0	25.1
9.0–9.9	182	−0.58	18.49	0.17	13.8	14.3	15.1	15.7	16.6	18.5	20.8	22.2	23.3	25.0	26.2
10.0–10.9	340	−0.67	19.19	0.17	14.4	14.9	15.7	16.3	17.2	19.2	21.6	23.1	24.3	26.1	27.4
11.0–11.9	369	−0.75	19.92	0.17	15.0	15.5	16.3	16.9	17.8	19.9	22.4	24.0	25.3	27.2	28.7
12.0–12.9	397	−0.84	20.63	0.17	15.6	16.1	16.9	17.5	18.5	20.6	23.3	24.9	26.2	28.3	29.9
13.0–13.9	316	−0.92	21.29	0.17	16.1	16.6	17.5	18.1	19.1	21.3	24.0	25.8	27.1	29.3	31.0
14.0–14.9	323	−0.98	21.85	0.17	16.6	17.1	18.0	18.6	19.6	21.9	24.6	26.4	27.8	30.2	31.9
15.0–15.9	276	−1.04	22.30	0.17	17.0	17.5	18.4	19.0	20.0	22.3	25.1	27.0	28.4	30.8	32.6
16.0–16.9	319	−1.07	22.65	0.17	17.3	17.8	18.7	19.3	20.4	22.6	25.5	27.4	28.8	31.2	33.1
17.0–17.9	405	−1.09	22.94	0.16	17.6	18.1	19.0	19.6	20.7	22.9	25.8	27.7	29.1	31.6	33.4
18.0–18.9	215	−1.08	23.24	0.16	17.8	18.4	19.2	19.9	20.9	23.2	26.1	28.0	29.5	31.9	33.8
19.0–19.9	485	−1.04	23.64	0.16	18.1	18.7	19.6	20.2	21.3	23.6	26.6	28.5	29.9	32.4	34.2
20.0–29.9	949	−0.99	24.23	0.16	18.6	19.1	20.1	20.7	21.8	24.2	27.2	29.1	30.6	33.0	34.8
30.0–39.9	178	−0.91	24.99	0.16	19.1	19.7	20.7	21.4	22.5	25.0	28.0	30.0	31.5	33.9	35.7
40.0–49.9	190	−0.81	25.84	0.16	19.7	20.3	21.3	22.1	23.3	25.8	29.0	31.0	32.4	34.9	36.7
50.0–59.9	292	−0.70	26.68	0.16	20.2	20.9	22.0	22.8	24.0	26.7	29.9	31.9	33.4	35.9	37.6
60.0–69.9	701	−0.57	27.48	0.16	20.7	21.4	22.6	23.4	24.7	27.5	30.8	32.8	34.3	36.8	38.5
70.0–79.9	433	−0.44	28.23	0.16	21.2	21.9	23.1	24.0	25.3	28.2	31.6	33.7	35.2	37.6	39.3
>80 years	70	−0.31	28.95	0.16	21.6	22.3	23.6	24.5	26.0	28.9	32.4	34.5	36.0	38.3	40.0

*LMS, least-mean-square algorithm; L, Box-Cox coefficient; M, median; S, coefficient of variation*.

**Table 5:** Percentiles for evaluating TMI from 5 to 80 years old based on age range for males.

**Age**	***n***	**L**	**M**	**S**	**P3**	**P5**	**P10**	**P15**	**P25**	**P50**	**P75**	**P85**	**P90**	**P95**	**P97**
5.0–5.9	149	−0.5	14.9	0.2	11.2	11.6	12.2	12.7	13.4	14.9	16.7	17.9	18.7	20.0	20.9
6.0–6.9	206	−0.5	14.7	0.2	11.0	11.4	12.0	12.5	13.2	14.7	16.5	17.6	18.4	19.7	20.6
7.0–7.9	279	−0.6	14.5	0.2	10.9	11.3	11.9	12.3	13.0	14.5	16.2	17.3	18.1	19.4	20.3
8.0–8.9	377	−0.7	14.2	0.2	10.8	11.1	11.7	12.1	12.8	14.2	16.0	17.0	17.8	19.1	20.0
9.0–9.9	302	−0.7	14.0	0.2	10.7	11.0	11.6	12.0	12.6	14.0	15.7	16.8	17.5	18.8	19.7
10.0–10.9	458	−0.8	13.8	0.2	10.5	10.9	11.4	11.8	12.4	13.8	15.4	16.5	17.3	18.5	19.4
11.0–11.9	524	−0.9	13.6	0.2	10.4	10.7	11.2	11.6	12.2	13.6	15.2	16.2	17.0	18.2	19.2
12.0–12.9	396	−0.9	13.4	0.2	10.3	10.6	11.1	11.5	12.1	13.4	14.9	16.0	16.7	18.0	18.9
13.0–13.9	420	−1.0	13.2	0.2	10.2	10.5	11.0	11.4	12.0	13.2	14.8	15.8	16.6	17.8	18.8
14.0–14.9	457	−1.1	13.2	0.2	10.2	10.5	11.0	11.4	11.9	13.2	14.7	15.7	16.5	17.8	18.7
15.0–15.9	524	−1.2	13.2	0.2	10.3	10.6	11.1	11.4	12.0	13.3	14.8	15.8	16.6	17.9	18.8
16.0–16.9	464	−1.2	13.4	0.2	10.5	10.8	11.2	11.6	12.2	13.4	15.0	16.0	16.7	18.1	19.1
17.0–17.9	777	−1.2	13.6	0.2	10.7	11.0	11.4	11.8	12.4	13.6	15.2	16.2	17.0	18.3	19.3
18.0–18.9	397	−1.2	13.8	0.2	10.9	11.2	11.7	12.0	12.6	13.8	15.4	16.5	17.3	18.6	19.6
19.0–19.9	467	−1.2	14.1	0.1	11.1	11.4	11.9	12.3	12.9	14.2	15.8	16.8	17.6	19.0	20.0
20.0–29.9	1,058	−1.2	14.5	0.1	11.4	11.8	12.3	12.6	13.2	14.5	16.2	17.2	18.0	19.4	20.4
30.0–39.9	111	−1.2	14.9	0.1	11.8	12.1	12.6	13.0	13.6	14.9	16.6	17.7	18.5	19.9	20.9
40.0–49.9	64	−1.2	15.4	0.1	12.1	12.5	13.0	13.4	14.0	15.4	17.1	18.1	19.0	20.4	21.4
50.0–59.9	117	−1.2	15.8	0.1	12.5	12.8	13.4	13.7	14.4	15.8	17.5	18.6	19.4	20.9	21.9
60.0–69.9	272	−1.2	16.1	0.1	12.8	13.1	13.7	14.1	14.7	16.2	17.9	19.0	19.9	21.3	22.3
70.0–79.9	203	−1.2	16.5	0.1	13.1	13.5	14.0	14.4	15.1	16.5	18.3	19.4	20.3	21.7	22.8
>80 years	48	−1.2	16.9	0.1	13.4	13.8	14.3	14.8	15.4	16.9	18.7	19.8	20.7	22.1	23.2

*LMS, least-mean-square algorithm; L, Box-Cox coefficient; M, median; S, coefficient of variation*.

**Table 6:** Percentiles for evaluating TMI from 5 to 80 years old based on age range for females.

**Age**	**n**	**L**	**M**	**S**	**P3**	**P5**	**P10**	**P15**	**P25**	**P50**	**P75**	**P85**	**P90**	**P95**	**P97**
5.0–5.9	137	0.15	14.73	0.16	10.8	11.3	12.0	12.5	13.2	14.7	16.4	17.4	18.0	19.1	19.8
6.0–6.9	220	0.02	14.49	0.16	10.7	11.1	11.8	12.3	13.0	14.5	16.2	17.1	17.8	18.9	19.6
7.0–7.9	302	−0.12	14.26	0.16	10.6	11.0	11.6	12.1	12.8	14.3	15.9	16.9	17.6	18.7	19.4
8.0–8.9	267	−0.26	14.08	0.16	10.5	10.9	11.5	11.9	12.6	14.1	15.7	16.7	17.4	18.6	19.4
9.0–9.9	182	−0.40	13.94	0.16	10.4	10.8	11.4	11.8	12.5	13.9	15.6	16.6	17.3	18.5	19.3
10.0–10.9	340	−0.53	13.87	0.16	10.4	10.8	11.4	11.8	12.5	13.9	15.5	16.6	17.3	18.5	19.4
11.0–11.9	369	−0.66	13.86	0.16	10.5	10.8	11.4	11.8	12.5	13.9	15.6	16.6	17.4	18.7	19.6
12.0–12.9	397	−0.78	13.93	0.16	10.6	10.9	11.5	11.9	12.5	13.9	15.7	16.7	17.5	18.9	19.9
13.0–13.9	316	−0.89	14.06	0.17	10.7	11.0	11.6	12.0	12.6	14.1	15.8	16.9	17.8	19.2	20.2
14.0–14.9	323	−0.97	14.21	0.17	10.8	11.2	11.7	12.1	12.8	14.2	16.0	17.2	18.0	19.5	20.6
15.0–15.9	276	−1.04	14.36	0.17	11.0	11.3	11.8	12.3	12.9	14.4	16.2	17.4	18.3	19.8	21.0
16.0–16.9	319	−1.07	14.49	0.17	11.1	11.4	12.0	12.4	13.0	14.5	16.3	17.6	18.5	20.1	21.3
17.0–17.9	405	−1.08	14.61	0.17	11.1	11.5	12.1	12.5	13.1	14.6	16.5	17.7	18.7	20.3	21.5
18.0–18.9	215	−1.06	14.76	0.17	11.2	11.6	12.2	12.6	13.3	14.8	16.7	17.9	18.8	20.5	21.7
19.0–19.9	485	−1.01	15.00	0.17	11.4	11.7	12.3	12.8	13.5	15.0	16.9	18.2	19.1	20.8	22.0
20.0–29.9	949	−0.94	15.41	0.17	11.7	12.0	12.7	13.1	13.8	15.4	17.4	18.7	19.6	21.3	22.5
30.0–39.9	178	−0.84	16.00	0.17	12.1	12.5	13.1	13.6	14.3	16.0	18.1	19.4	20.4	22.0	23.2
40.0–49.9	190	−0.73	16.68	0.17	12.5	12.9	13.6	14.1	14.9	16.7	18.8	20.2	21.2	22.8	24.0
50.0–59.9	292	−0.60	17.38	0.17	13.0	13.4	14.1	14.7	15.5	17.4	19.6	21.0	22.0	23.7	24.8
60.0–69.9	701	−0.46	18.06	0.17	13.4	13.9	14.6	15.2	16.1	18.1	20.3	21.7	22.8	24.4	25.6
70.0–79.9	433	−0.31	18.71	0.17	13.7	14.3	15.1	15.7	16.7	18.7	21.1	22.5	23.5	25.2	26.3
>80 years	70	−0.17	19.35	0.17	14.1	14.7	15.6	16.2	17.2	19.4	21.8	23.2	24.3	25.9	27.0

*LMS, least-mean-square algorithm; L, Box-Cox coefficient; M, median; S, coefficient of variation*.

The TMI value for 50th percentile moved curvi-linearly from ages 5.0–5.9 to 80 years old. For example, the values decreased slightly until ages 11.0–11.9, approximately. Then, these values remained stable until ages 16.0–16.9. Finally, TMI increased linearly until 80 years of age. The differences between both sexes began appearing from 12.0 to 12.9 years old onwards. It was greater in females when compared to males (from ~0.6 to ~2.5 kg/m^3^) until approximately age 80.

## Discussion

The results from this study indicate that during all stages of life, the regional population of the Maule showed similar trajectories for weight, height, and BMI to those of the American NCHS (8). However, differences occurred during childhood where the children from this study showed increased weight and BMI than the references. Nevertheless, during adolescence, the NCHS references (8) showed higher values until around ages 70–79, respectively. In addition, we highlight that children and adolescents of both sexes in Maule (5–19 years of age) presented higher BMI values in relation to those of the WHO in all age ranges (from 5 to 19 years of age).

During early ages, height was relatively similar to the NCHS references (8). However, during adolescence, differences began to appear where the population of the Maule was shorter until approximately age 80.

Therefore, these findings are relevant for the regional population of the Maule because they confirm the anthropometric differences between populations, especially during the growth stages. Previous research in Chile (13, 14) and other neighboring countries have also highlighted such discrepancies (24, 25). However, during adulthood, between the ages of 20 to 59 years old, some studies carried out in China (26) and Colombia (27) showed values relatively lower in weight, height, and BMI than those of the Maule population. Nevertheless, other studies conducted in Canada (28) and the United States (29) have reported BMI values relatively similar to those described in this present research.

During old age, from 60 until age 80 years old, the BMI values of the Maule regional population were relatively similar to those reported in Brazil, Portugal, and Mexico (30–32) and even with the Chilean reference proposed for older adults (33).

One aspect that caught the researchers' attention was that the BMI of the Maule population did not decrease at age 80 as it did in other studies (8, 31, 32). This could be due, perhaps, to the loss of height with age. This translates into a significant increase in BMI (34). This pattern of decrease was observed in both sexes commencing at age 40 onwards.

These variations in the anthropometric profile between world populations from childhood to old age reflect genetic and environmental differences throughout life. For example, environmental factors tend to affect genetic potential and health differences in populations (9), including socio-demographic, dietary, and lifestyle factors (35). These could be involved in the differences observed. Although it is not ruled out that, during the last decades, Chile has faced an extremely rapid nutritional transition (36), which is congruent with the rapid economic growth observed between 1980 and 2014 (37), it is currently characterized as a post-transitional country, where the nutritional status of the population varies significantly according to sex, socioeconomic level and ethnicity (38).

In this context, an urgent need exists to gather additional measurements to provide a more holistic understanding of the anthropometric status of a population (39). Since the prevalence of underweight and overweight vary widely from one population to another (40), it is important to collect more data, especially if references proposed for other socio-cultural groups are used. These characteristics described deserve to be compared with reference studies as was done in this study, so this information could provide greater opportunities for comparison with other populations at the national and international level.

In this sense, based on the differences observed in this study and others, percentiles were developed to evaluate nutritional status from childhood to old age for the Maule Region (Chile). These were based on BMI and TMI by age and sex.

These results could help with the interpretation of the anthropometric differences and patterns in the phenotypical changes during growth and aging. This, during the growth and development stages, this information could facilitate evaluation of the nutritional state and serve to help monitor physical growth trajectories of children and adolescents (14).

During adulthood, these findings could help determine possible altered nutritional conditions that could be used as indicators for metabolic risk factors, especially when they are associated with overweight and obesity (41). In addition, nutrition and health, in general, in this stage are important for this group since adults are responsible for helping economically the rest of society (40).

In general, some common medical afflictions exist in old age related to aging and nutritional disorders that can be risk factors for older adults (42). For example, important changes occur in body composition expressed as the increase in fat mass, distribution of body fat, and the loss of muscle mass (43). These changes have adverse effects on functional ability, quality of life, and survival (44).

As a result, the researchers for this study used the LMS method to develop percentiles for BMI and TMI. This information may serve to complement the national (14, 33) and international (2, 7, 10, 45) references. A number of these were divided and directed to determined specific groups.

In essence, the references are data that are based on cross-sectional evaluations of a well-defined population (9). On the one hand, their interpretation needs to be oriented toward universal characteristics and human growth variables in order to research the links between growth, health, and nutritional state. On the other hand, it is necessary to evaluate thoroughly whether the international references are appropriate for all individuals, or whether sometimes specific references may be beneficial for a population (46).

Consequently, the applicability and tracking of the BMI trajectory during childhood, adolescence, and adulthood has demonstrated to be a satisfactory index for estimating body adiposity status once the changes body weight (47, 48), metabolic risks, and obesity (49) have been taken into account. In addition, it has been used traditionally as an indicator of obesity and for predicting health problems (50).

On the one hand, the use and applicability of TMI during the growth stage may be considered as a more appropriate tool to evaluate and classify nutritional status (underweight and obesity) for children and adolescents (51). A number of recent studies have reported that the TMI is more accurate than BMI for predicting body adiposity levels (17, 52) and for classifying overweight and obesity (53). It may also be used as a marker for metabolic syndromes (54) in diverse populations.

The cut-off points for this study were based on criteria adopted by the CDC (9) and the NCHS (8, 12). These need to be interpreted as references since they describe growth and nutritional status of an individual, and they provide a common foundation for comparing populations without making inferences about their significance (55). In addition, they may serve to formulate health policies, plan interventions, and supervise their efficiency, respectively (56).

We suggest the use of the Z-score for children and adolescents from 5 to 18 years old. This can be calculated using the formula:


$$\begin{array}{l} Z=\frac{{\left(\frac{BMI}{M}\right)}^{L}-1}{L\;x\;S}\; \end{array}$$

Where the values of L, M, and S refer to age and sex of each child and adolescent in keeping with the references in the literature. On the other hand, from 19 years of age onwards, the cut-off points described above (<10th percentile low, 10th to 85th percentile normal, 85th to 95th percentile overweight and >95th percentile obese) can be used.

The present study has several strengths. The researchers in this study used 15,436 subjects (8,070 men and 7,366 women), spanning most life stages from infancy to senescence. This is the first research study of its kind carried out in Chile and Latin America. Moreover, the anthropometric variables were evaluated by only one experienced and well trained research team. The results may be used as a baseline for future comparisons for verifying secular tendencies. They may also help serve to create a national anthropometric standard.

This study had some limitations that need to be acknowledged. For example, the children (minors) <5.0 years old could not be included in the study due to lack of access to them. This limited us in evaluating the classic standard anthropometric variables of weight and height and their corresponding relationships (weight by height squared and weight by height cubed). The sample selection was non-probabilistical, so this procedure could limit the generalization of the results to other socio-cultural contexts in the country. Furthermore, it was not possible to collect socio-economic information, dietary habits, ethnicity and physical activity levels. Moreover, each age group considered in this study may be affected by different environmental, sociocultural, and political influences throughout their lives, especially during the military period from 1973 to 1990 and since the early 1980s with the rapid socioeconomic growth (37).

Other researchers should consider the present results with caution, as the mentioned components should be evaluated in the future, and even project the use of GWAS for more precise comparisons in terms of genetic variation.

In conclusion, by determining differences between the American NCHS references with the anthropometric variables of weight and height and in BMI, reference percentiles were developed for assessing the nutritional status for the regional population of the Maule (Chile). The results suggest using BMI and TMI by age and sex as a non-invasive tool for detecting individuals at risk of being underweight, overweight, and obese. Finally, these results may be used in clinical and epidemiological contexts to assess individuals, age 5–80 years old.

## Data Availability Statement

The raw data supporting the conclusions of this article will be made available by the authors, without undue reservation.

## Ethics Statement

The studies involving human participants were reviewed and approved by the Ethics Committee from Universidad Autonoma de Chile (238/2014). Written informed consent to participate in this study was provided by the participants' legal guardian/next of kin.

## Author Contributions

MC-B, RG-C, AM, and EL conceived and designed the study. MC-B, RG-C, RV-E, LC, and LU-A performed the experiments. AM and EL contributed reagents/materials/analysis tools. RG-C, MC-B, and WC-B performed the statistical analysis. MC-B, RG-C, AM, EL, and RV-E drafted the manuscript. CA participated in the drafting, translation, and correction of the manuscript. All authors edited and revised the manuscript with critical feedback given.

## Funding

This work was supported by the National Commission of Science and Technology, CONICYT, Chile under Grant (Number 1141295).

## Conflict of Interest

The authors declare that the research was conducted in the absence of any commercial or financial relationships that could be construed as a potential conflict of interest.

## Publisher's Note

All claims expressed in this article are solely those of the authors and do not necessarily represent those of their affiliated organizations, or those of the publisher, the editors and the reviewers. Any product that may be evaluated in this article, or claim that may be made by its manufacturer, is not guaranteed or endorsed by the publisher.

## References

[1] Bhattacharya A Pal B Mukherjee S Roy SK. Assessment of nutritional status using anthropometric variables by multivariate analysis. BMC Public Health. (2019) 19:1045. 10.1186/s12889-019-7372-2 31382936 PMC6683359

[2] World Health Organization (WHO). Improvement of Nutritional Status of Adolescents (2002). Available online at: http://apps.searo.who.int/pds_docs/B3526.pdf (accessed February 5, 2014)

[3] Bhutta ZA Salam RA. Global nutrition epidemiology and trends. Ann Nutr Metab. (2012) 61: 19–27. 10.1159/000345167 10.1159/000345167 23343944

[4] Dwyer JT Gallo JJ Reichel W. Assessing nutritional status in elderly patients. Am Fam Physician. (1993) 47:613–20. 8434552

[5] Simko MD Cowell C Gilbride JA. Nutrition Assessment: A Comprehensive Guide for Planning Intervention. 2nd ed. Gaithersburg, MD: Aspen Publishers (1995).

[6] Bharati S Pal M Bhattacharya BN Bharati P. Prevalence and causes of chronic energy deficiency and obesity in Indian women. Hum Biol. (2007) 79:395–412. 10.1353/hub.2007.0048 18075004

[7] De Onis M Habicht JP. Anthropometric reference data for international use: recommendations from a WHO expert committee. Food Nutr Bull. (1997) 18:179–89. 10.1177/156482659701800204 8839517

[8] Fryar CD Gu Q Ogden CL. Anthropometric reference data for children and adults: United States, 2007–2010. National Center for Health Statistics. Vital Health Stat. (2012) 11:1–28. Available online at: https://www.cdc.gov/nchs/data/series/sr_11/sr11_252.pdf (accessed December 18, 2020). 25204692

[9] Kuczmarski R Ogden C Grummer-Strawn L Flegal KM Guo SS Wei R. CDC Growth Charts: United States. Advance Data From Vital and Health Statistics. Hyattsville, MD: US Department of Health and Human Services (2000). 11183293

[10] WHO Multicentre Growth Reference Study Group. WHO Child Growth Standards: Length/Height-for-Age, Weight-for-Age, Weight-for-Length, Weight for-Height and Body Mass Index-for-Age: Methods and Development. Geneva: World Health Organization (2006).

[11] de Onis M Onyango AW Borghi E Siyam A Nishida C Siekmann J. Development of a WHO growth reference for school-aged children and adolescents. Bull World Health Organ. (2007) 85:660–7. 10.2471/BLT.07.043497 18026621 PMC2636412

[12] Fryar CD Gu Q Ogden CL Flegal KM. Anthropometric reference data for children and adults: United States, 2011–2014. Vital Health Stat. (2016) 3:39. 28437242

[13] Gómez-Campos R Andruske CL Hespanhol J Sulla-Torres J Arruda M Luarte-Rocha C Cossio-Bolaños M. Waist circumferences of Chilean students: comparison of the CDC-2012 standard and proposed percentile curves. Int J Environ Res Public Health. (2015) 12:7712–24. 10.3390/ijerph120707712 26184250 PMC4515686

[14] Gomez-Campos R Arruda M Andruske CL Leite-Portella D Pacheco-Carrillo J Urra-Albornoz C. Physical growth and body adiposity curves in students of the Maule Region (Chile). Front Pediatr. (2019) 7:323. 10.3389/fped.2019.00323 31448248 PMC6691029

[15] Prista A Maia JA Damasceno A Beunen G. Anthropometric indicators of nutritional status: implications for fitness, activity, and health in school-age children and adolescents from Maputo, Mozambique. Am J Clin Nutr. (2003) 77:952–9. 10.1093/ajcn/77.4.952 12663297

[16] Gorstein J. Assessment of nutritional status: effects of different methods to determine age on the classification of under nutrition. Bull WHO. (1989) 67:143–50. 2635590 PMC2491244

[17] Peterson CM Su H Thomas DM Heo M Golnabi AH Pietrobelli A. TriPonderal mass index vs body mass index in estimating body fat during adolescence. JAMA Pediatr. (2017) 171:629–36. 10.1001/jamapediatrics.2017.0460 28505241 PMC5710345

[18] Wang X Dong B Ma J Song Yi Zou Z Luke A. Role of tri-ponderal mass index in cardio-metabolic risk assessment in children and adolescents: compared with body mass index. Int J Obes. (2020) 44:886–94. 10.1038/s41366-019-0416-y 31332274

[19] Ministerio de desarrollo social Chile (MDS),. Informe de Desarrollo Social. (2018). Available online at: http://www.desarrollosocialyfamilia.gob.cl/storage/docs/Informe_de_Desarrollo_Social_2018.pdf (accessed December 18, 2020).

[20] United Nations Development Programme (UNDP). Human Development Report 2019. Beyond Income, Beyond Averages, Beyond Today: Inequalities in Human Development in the 21st Century. New York, NY: One United Nations (2019). Available online at: http://hdr.undp.org/sites/default/files/hdr2019.pdf (accessed December 18, 2020).

[21] Ross WD Marfell-Jones MJ. Kinanthropometry. In: MacDougall JD Wenger HA Geeny HJ editor. Physiological Testing of Elite Athlete, Vol 223. London: Human Kinetics. (1991). p. 308–14.

[22] Cole TJ Bellizzi MC Flegal KM Dietz WH. Establishing a standard definition for child overweight and obesity worldwide: international survey. BMJ. (2000) 320:1240–43. 10.1136/bmj.320.7244.1240 10797032 PMC27365

[23] Pan H Cole TJ. LMS Chartmaker. (2006). Available online at: http://www.healthforallchildren.co.uk (accessed March 28, 2015).

[24] Campos RG de Arruda M Hespanhol JE Camargo C Briton RM Cossio-Bolanõs MA. Referencial values for the physical growth of school children and adolescents in Campinas, Brazil. Ann Hum Biol. (2015) 42:62–9. 10.3109/03014460.2014.927920 24981888

[25] Cossio-Bolaños M Gómez-Campos R Andruske C Viveros-Flores A Luarte C Olivares P. Physical growth, biological age, and nutritional transitions of adolescents living at moderate altitudes in Peru. Int J Environ Res Public Health. (2015) 12:12082–94. 10.3390/ijerph121012082 26404334 PMC4626956

[26] Zeng Q He Y Dong S Zhao X. Optimal cut-off values of BMI, waist circumference and waist height ratio for defining obesity in Chinese adults. Br J Nutr. (2014) 112:1735–44. 10.1017/S0007114514002657 25300318

[27] Ramírez-Vélez R Correa-Bautista JE Martínez-Torres J Méneses-Echavez JF González-Ruiz K González-Jiménez E. LMS tables for waist circumference and waist-height ratio in Colombian adults: analysis of nationwide data 2010. Eur J Clin Nutr. (2016) 70:1189–96. 10.1038/ejcn.2016.46 27026425 PMC5056989

[28] Galloway T Chateau-Degat ML Egeland GM Young T. K. Does sitting height ratio affect estimates of obesity prevalence among Canadian Inuit? Results from the 2007-2008 Inuit Health Survey. Am J Hum Biol. (2011) 23:655–63. 10.1002/ajhb.21194 21681849

[29] Chumlea WC Guo SS Zeller CM Reo N.V. Siervogel R.M.. Total body water data for white adults 18 to 64 years of age: the Fels Longitudinal Study. Kidney Int. (1999) 56:244–52. 10.1046/j.1523-1755.1999.00532.x 10411699

[30] Segheto W Coelho FA Cristina Guimarães da Silva D Curi Hallal P Bouzas Marins JC Queiroz Ribeiro A. Validity of body adiposity index in predicting body fat in Brazilians adults. Am J Hum Biol. (2017) 29:22901. 10.1002/ajhb.22901 27502080

[31] Marques EA Baptista F Santos R Vale S Santos D Silva AM. Normative functional fitness standards and trends of Portuguese older adults: cross-cultural comparisons. J Aging Phys Act. (2014) 22:126–37. 10.1123/japa.2012-0203 23538513

[32] López-Ortega M Arroyo P. Anthropometric characteristics and body composition in Mexican older adults: age and sex differences. Br J Nutr. (2016) 115:490–9. 10.1017/S0007114515004626 26597049

[33] Santos JL Albala C Lera L Gaercia C Arroyo M Perez-Bravo F. Anthropometric measurements in the elderly population of Santiago, Chile. Nutrition. (2004) 20:452–7. 10.1016/j.nut.2004.01.010 15105033

[34] Gavriilidou NN Pihlsgard M Elmstahl S. High degree of BMI misclassification of malnutrition among Swedish elderly population: age-adjusted height estimation using knee height and demispan. Eur J Clin Nutr. (2015) 69:565–71. 10.1038/ejcn.2014.183 25205322 PMC4424802

[35] Sanchez-Garcia S Garcia-Pena C Duque-Lopez MX Juarez-Cedillo T Cortes-Nunez AR Reyes-Beaman S. Anthropometric measures and nutritional status in a healthy elderly population. BMC Public Health. (2007) 7:2. 10.1186/1471-2458-7-2 17201919 PMC1769489

[36] Vio F Albala C Kain J. Nutrition transition in Chile revisited: mid-term evaluation of obesity goals for the period 2000–2010. Public Health Nutr. (2008) 11:405–12. 10.1017/S136898000700050X 17617931

[37] Gammage S Alburquerque T Durán G. Poverty, Inequality and Employment in Chile. Geneva: International Labour Office (ILO), Conditions of Work and Employment Series (2014)

[38] Mujica-Coopman MF Navarro-Rosenblatt D López-Arana S Corvalán C. Nutrition status in adult Chilean population: economic, ethnic and sex inequalities in a post-transitional country. Public Health Nutr. (2020) 23:s39–50. 10.1017/S1368980019004439 32131930 PMC10200670

[39] Gavriilidou N Pihlsgård M Elmståhl S. Anthropometric reference data for elderly Swedes and its disease-related pattern. Eur J Clin Nutr. (2015) 69:1066–75. 10.1038/ejcn.2015.73 25990690 PMC4559758

[40] de Onis M Habicht JP. Anthropometric reference data for international use: recommendations from a World Health Organization Expert Committee. Am J Clin Nutr. (1996) 64:650–8. 10.1093/ajcn/64.4.650 8839517

[41] James WPT Chunming C Inoue S. Appropriate Asian body mass indices? Obes Rev. (2002) 3:139. 10.1046/j.1467-789X.2002.00063.x 12164464

[42] Dey DK Rothenberg E Sundh V Bosaeus I Steen B. Body mass index, weight change and mortality in the elderly. A 15 y longitudinal population study of 70 y olds. Eur J Clin Nutr. (2001) 55:482–92. 10.1038/sj.ejcn.1601208 11423925

[43] Pagotto V Silveira EA. Methods, diagnostic criteria, cutoff points, and prevalence of sarcopenia among older people. Sci World J. (2014) 2014:231312. 10.1155/2014/231312 25580454 PMC4280495

[44] Beaudart C Zaaria M Pasleau F Reginster JY Bruyère O. Health outcomes of sarcopenia: a systematic review and meta-analysis. PLoS ONE. (2017) 12:e0169548. 10.1371/journal.pone.0169548 28095426 PMC5240970

[45] Kuczmarski MF Kuczmarski RJ Najjar M. Descriptive anthropometric reference data for older Americans. J Am Diet Assoc. (2000) 100:59–66. 10.1016/S0002-8223(00)00021-3 10646006

[46] Blackwell A Urlacher S Beheim B Rueden C Jaeggi A Stieglitz J. Growth references for Tsimane forager-horticulturalists of the Bolivian Amazon. Am J Phys Anthropol. (2017) 162:441–61. 10.1002/ajpa.23128 28218400 PMC5321633

[47] Rolland-Cachera MF Sempé M Guilloud-Bataille M Patois E Péquignot-Guggenbuhl F Fautrad V. Adiposity indices in children. Am J Clin Nutr. (1982) 36:178–84. 10.1093/ajcn/36.1.178 7091028

[48] Giudici KV Rolland-Cachera MF Gusto G Goxe D Lantieri O Hercberg S. Body mass index growth trajectories associated with the different parameters of the metabolic syndrome at adulthood. Int J Obes. (2017) 41:1518–25. 10.1038/ijo.2017.119 28529329

[49] Eriksson JG. Early growth and coronary heart disease and type 2 diabetes: findings from the Helsinki Birth Cohort Study (HBCS). Am J Clin Nutr. (2011) 94(6 Suppl):1799S−802S. 10.3945/ajcn.110.000638 21613556

[50] Savva SC Tornaritis M Savva ME. Waist circumference and waist-to-height ratio are better predictors of cardiovascular disease risk factors in children than body mass index. Int J Obes Relat Metab Disord. (2000) 24:1453–8. 10.1038/sj.ijo.0801401 11126342

[51] Carrascosa A Yeste D Moreno-Galdó A Gussinye M Ferrandez A Clemente M. Fernandez-Cancio M. Índice de masa corporal e índice de masa triponderal de 1453 niños no obesos ni malnutridos de la generación del milenio Estudio longitudinal de Barcelona. An Pediatr. (2018) 89:137–43. 10.1016/j.anpedi.2017.12.016 29478880

[52] De Lorenzo A Romano L Di Renzo L Gualtieri P Salimei C Carrano E. Triponderal mass index rather than body mass index: an indicator of high adiposity in Italian children and adolescents. Nutrition. (2019) 60:41–7. 10.1016/j.nut.2018.09.007 30529185

[53] Moselakgomo VK Van Staden M. Diagnostic accuracy of tri-ponderal mass index and body mass index in estimating overweight and obesity in South African children. Afr J Prim Health Care Fam Med. (2019) 11:e1–7. 10.4102/phcfm.v11i1.1949 31478739 PMC6739532

[54] Khoshhali M Heidari-Beni M Qorbani M Motlagh-Mohammad E Ziaodini H Heshmat R. Tri-ponderal mass index and body mass index in prediction of pediatric metabolic syndrome: the CASPIAN-V study. Arch Endocrinol Metab. (2020) 64:171–8. 10.20945/2359-3997000000206 32236304 PMC10118948

[55] Turck D Michaelsen KF Shamir R Braegger C Campoy C Colomb V. World Health Organization 2006 child growth standards and 2007 growth reference charts: a discussion paper by the committee on nutrition of the European Society for Pediatric Gastroenterology, Hepatology, and Nutrition. J Pediatr Gastroenterol Nutr. (2013) 57:258–64. 10.1097/MPG.0b013e318298003f 23880630

[56] De Onis M. Growth curves for school-age children and adolescents. Ind Pediatr. (2009) 46:463–5. 19556656

